# Effects of Storage Time on Total Protein and Globulin Concentrations in Bovine Fresh Frozen Plasma Obtained for Transfusion

**DOI:** 10.1155/2015/752724

**Published:** 2015-02-12

**Authors:** D. Proverbio, E. Spada, L. Baggiani, G. Bagnagatti De Giorgi, N. Roggero, A. Belloli, D. Pravettoni, R. Perego

**Affiliations:** Dipartimento di Scienze Veterinarie per la Salute, la Produzione Animale e la Sicurezza Alimentare, Università degli Studi di Milano, Via Celoria 10, 20133 Milano, Italy

## Abstract

To evaluate the effects of storage conditions on total protein (TP) and globulin fractions in fresh frozen bovine plasma units prepared and stored for transfusion, TP and globulin fractions were evaluated in fresh plasma and at 1 month and 6 and 12 months after blood collection in plasma stored at −20°C. Significant differences in concentrations were found in the median concentration of total protein (*P* = 0.0336), between 0 months and 1 month (*P* = 0.0108), 0 and 6 months (*P* = 0.0023), and 0 and 12 months (*P* = 0.0027), in mean concentration (g/dL) of albumin (*P* = 0.0394), between 0 months and 1 month (*P* = 0.0131), 0 and 6 months (*P* = 0.0035), and 0 and 12 months (*P* = 0.0038), and beta-2 fraction (*P* = 0.0401), between 0 and 6 months (*P* = 0.0401) and 0 and 12 months (*P* = 0.0230). This study suggests that total gamma globulin concentration in bovine frozen plasma is stable for 12 months at −20°C. Total protein, ALB, and beta-2 fraction have significantly different concentrations (g/dL) when compared to prestorage. This study has shown IgG protein fraction stability in bovine fresh frozen plasma collected for transfusion; therefore, bovine fresh frozen plasma seems to be suitable for the treatment of hypogammaglobulinemia (failure of passive transfer) in calves when stored for 12 months at −20°C.

## 1. Introduction

Fractionated blood products in farm animal transfusion medicine include packed RBCs, platelet-rich plasma (PRP), leukocyte-rich plasma, normal bovine plasma, and hyperimmune serum. Of these, only two are frequently used in cows and are bovine plasma and hyperimmune serum [1, 2].

In ruminant transfusion practice, fresh frozen plasma (FFP) can be used for treatment of hypogammaglobulinemia (failure of passive transfer) in calves [3, 4]. Calves are born hypogammaglobulinemic and require colostrum to supply immunoglobulins during the neonatal period. Neonatal calf health is largely dependent on the ingestion and absorption of maternally derived antibodies via colostrum consumption [5]. Failure of passive transfer (inadequate circulating IgG concentration) in calves is a common condition that predisposes calves to increased morbidity and mortality and there is a link between low serum globulins and the incidence of infectious diseases [6]. The importance of the ingestion and absorption of colostral immunoglobulins on morbidity, mortality, growth, and future productivity of dairy calves has been described [7]. Calves with inadequate passive transfer of colostral immunoglobulins have an increased risk of death during the first 3 months after birth [8], a decreased rate of weight gain [9], and a decreased survival rate until the end of the first lactation [10, 11].

Many studies that have evaluated bovine serum administration have shown this to be an effective source of exogenous passive Ig for newborn calves [2, 5, 6, 12–15].

In dogs and mice plasma proteins appear to be stable during storage when frozen [16, 17]. Previous studies showed that there was no significant change in total protein and globulin fractions, compared with baseline values, in samples of frozen animal plasma stored for up to 7 days [16–18]. Most of these studies, however, were carried out on plasma obtained from blood, which had been collected using a needle and syringe, and transferred into lithium-heparin tubes. These conditions are quite different from those used for preparation of plasma intended for transfusion purposes which is typically separated from blood collected using a closed system into bags containing citrate-phosphate-dextrose-adenine-1 (CPDA-1) anticoagulant and stored in plastic bags at −20°C. Furthermore, there is a lack of information about the protein stability of frozen bovine plasma.

The aim of this study was to evaluate if the bovine plasma obtained with anticoagulant CPDA-1 could be electrophoresed and to evaluate the effects of storage conditions on TP and globulin fractions in fresh frozen bovine plasma units prepared and stored for use in transfusion.

## 2. Materials and Methods

### 2.1. Blood Collection

This prospective study was performed as an internal quality control at the Veterinary Transfusion Unit Blood Bank of University of Milan (REV). Blood was collected from 20 healthy lactating adult Holstein Friesian donors. Before and after blood collection all cows were given a standard physical examination [19]. A total volume of 4 L of blood was collected from each cow. The protocol for this study was approved by the Institutional Ethical Committee for Animal Care at University of Milan (http://www.unimi.it/cataloghi/comitato_etico/CE_19dic2012_verbale.pdf). A closed-collection system was used, consisting of sterile human 450 mL blood bags (TERUMO CPDA-1 triple blood bag, GRIFOLS, Italy) containing 20 mL of citrate-phosphate-dextrose-adenine-1 (CPDA-1) anticoagulant, used to collect blood from each cow. From each cow 8 bags of whole blood were collected. Whole blood was collected in a standard fashion from each cow by jugular venipuncture, using a 16-gauge needle attached to a triple-bag closed-collection system on a blood mixer. The closed-collection system consisted of a primary bag containing 63 mL of citrate phosphate-double dextrose solution as anticoagulant, an additive bag that contained 100 mL of additive solution (SAG-Mannitol), and 1 empty satellite bag.

Sterile 450 mL whole blood bags were centrifuged in refrigerated centrifuge (ROTIXA 50RS, Hettich, Germany) at 900 g for 15 minutes at 4°C. A manual plasma extractor (Separation Stand Teruflex ACS-201, United States) was then used to immediately generate 1 bag of 300 mL of plasma from each unit of blood. Small hand sealer clips were used to create 4 segments of tubing that each contained approximately 1 mL of plasma for each satellite bag.

One segment was used immediately as fresh plasma to obtain the baseline concentrations of plasma total protein (TP) and albumin (ALB) concentrations and plasma protein electrophoresis. Both bags of plasma and the remaining segments were immediately frozen at −20°C and stored in a controlled-temperature blood bank refrigerator (EMOTECA 250, Fiocchetti CO., Italy), where the temperature was consistently maintained at −20°C.

Plasma samples for all analyses were taken from tubing segments at 1 month and 6 and 12 months; after blood collection, one segment for each bag of plasma was thawed at room temperature (37°C).

### 2.2. Analytic Procedures

Total protein and ALB concentration and plasma protein fractions were determined for each fresh aliquot and for each frozen aliquot thawed after 1 month and 6 and 12 months of storage. Total protein plasma concentration was measured by spectrophotometry using the colorimetric biuret method (previously validated for bovines) [20] on a Cobas Mira Classics Roche automated chemistry analyzer (Roche S.p.A., Mannheim, Germany; within-run precision 0.48%; between-run precision 0.88%) that required 10 *μ*L of plasma. Commercial multiparameter human sera were used to calibrate the machine and to act as controls (Calibrator and Control Serum, Ben Srl, Milan, Italy). Plasma albumin concentration was measured by spectrophotometry using BCG colorimetric method (previously validated for bovines) [20] on a Cobas Mira Classics Roche automated chemistry analyzer (Roche S.p.A., Mannheim, Germany; within-run precision 1.65%; between-run precision 0.96%) with 10 *μ*L of plasma.

Protein fractions were analyzed using a semiautomatic agarose gel electrophoresis (AGE) system (Hydrasys, Sebia PN 1210, Issy-les-Moulineaux, France) (previously validated for bovine). Plasma was electrophoresed for 7 minutes at 33-volt hours and stained with diluted Amidoschwarz dye at pH 2 (4 g/L Amidoschwarz dye and 6.7% ethylene glycol). The AGE procedure was performed according to the manufacturer's instructions, and commercial human serum was used as the control (normal control serum, Sebia, Evry, France). Using the computer software Phoresis for Windows 2000 or XP Pro (Sebia), the electrophoretic curve for each sample was displayed. Protein fractions were determined based on the different percentage of optical absorbance and the absolute concentration in g/dL was automatically calculated from the total serum protein concentration. The same operator analyzed all samples. Albumin to globulin (A/G) ratios were also calculated. The percentage of variation observed between mean values of proteins analyzed in fresh and frozen/thawed sera was calculated for each protein fraction.

### 2.3. Statistical Analyses

The data distribution was evaluated with D'Agostino Pearson normality test. The Kruskal-Wallis test and paired *t*-test or the Mann-Whitney *U* test were used to compare results for fresh and frozen plasma at the different times of storage (MedCalc Software, version 12.7.8.0, Mariakerke, Belgium). Significance was set at *P* < 0.05.

## 3. Results

Albumin, alpha-1 fraction, expressed in g and as %, and A/G ratio were normally distributed. Mean, median, and 95% confidence interval of concentrations of total protein and ALB and alpha-1, alpha-2, beta-1, beta-2, and gamma globulin fractions concentrations, evaluated at different time of storage, are reported in Table 1. Concentrations (g/dL) of TP, ALB, and beta-2 fractions show significantly different increase from fresh and frozen plasma.

The results indicate that plasma concentrations (g/dL) of TP and ALB change after 1 month of storage at −20°C, whereas concentrations of beta-2 protein fractions were stable for 6 months at −20°C.

The significant effects of storage time on mean concentration of TP and on the concentration of protein fractions with the percentage of variation observed in fresh and frozen/thawed sera for each protein fraction are reported in Table 2.

**Figure 1:**
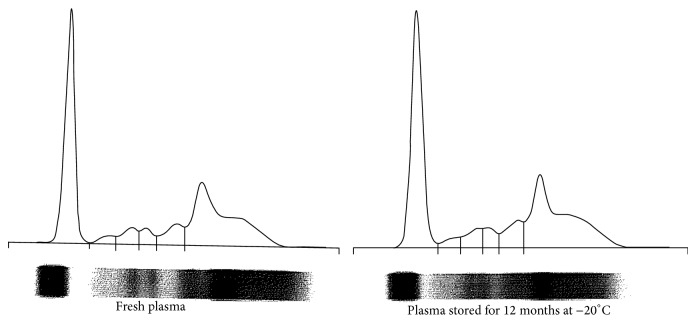
Representative serum protein electropherograms using agarose gel electrophoresis in bovine plasma obtained for transfusion purposes (anticoagulant CPDA-1) in fresh plasma sample and plasma sample stored frozen (−20°C) for 12 months. Note the appearance of 6 peaks: albumin (ALB) and alpha-1, alpha-2, beta-1, beta-2, and gamma globulin fractions.

On electropherograms there were 6 protein fractions: albumin, alpha-1, alpha-2, beta-1, beta-2, and gamma globulins. All samples analyzed at the different time of storage had comparable electrophoretic patterns (Figure 1).

## 4. Discussion

There is a paucity of information about serum protein profiles in cattle. Most of the data were collected years ago, with different support media [20–23]. In our study the plasma TP and ALB and concentrations of protein fractions, obtained by agarose gel support media, were comparable with those reported by Tóthová et al. [24] in a recent study, while the mean values of alpha globulin and gamma globulins are, respectively, lower and higher than values reported by Kaneko [25].

In accordance with other studies [24, 26], agarose-supporting matrix permitted identification of 6 electrophoretic bands with good resolution in all fresh and frozen samples. All samples had similar electropherograms, with comparable electrophoretic patterns. In our study CPDA-1 was used as the anticoagulant, while in previous studies serum or plasma was obtained using lithium-heparin anticoagulant. Our results suggest that the type of anticoagulant does not affect the resolution of the electrophoretic method.

Serum proteins have been reported to remain stable in storage [16–18]. The effect of duration and temperature of storage on canine plasma and serum constituents has been documented [16]. Most analytes show no or very mild change in canine plasma [16] and concentrations of glucose and total proteins in human samples have been shown to be unaffected by repeated freeze-thaw cycles [27]. Our results concur with results of previous studies that evaluated the effects of freezing and storage on routine assays of total protein and protein fractions in other animals [16–18]. In those studies, however, blood samples were collected by means of direct venipuncture, using a syringe and needle, and placed in glass or plastic tubes (either empty or containing lithium-heparin), conditions which do not mimic those used for obtaining and storing plasma for transfusion.

In our study, when the value of protein fractions was expressed as a percentage of TP, no difference was found between fresh plasma and thawed plasma at any storage time. However, when the values were expressed in g/dL, significant differences were seen, with a number of analytes occurring at higher concentrations in frozen/thawed serum than in fresh serum, for example, mean concentration of TP between 0 months and 1 month (6,4%), 0 and 6 months (6.4%), and 0 and 12 months (3.5%); ALB concentration between 0 months and 1 month, 0 and 6 months, and 0 and 12 months (7.6%); and beta-2 concentration between 0 and 6 months and 0 and 12 months (7.5% and 13.2%, resp.). Reynolds et al. [17] reported similar results in a study examining effects on canine plasma protein after repeated freeze-thaw cycles. In our study the change in the mean values exceeded “accepted value of within subject biological variation” and “total error allowed for people” [28], and in the case of beta globulin the percentage of variation exceeded 10%. In accordance with the interpretation of Cray et al. [18] we accepted that differences greater than 10% of the minimal difference are likely to have clinical significance in the interpretation of the biochemical analysis.

The comparison of analysis of bovine plasma with human ranges of biological variation may be erroneous, and without species-specific ranges of biological variability, definitive conclusions cannot be drawn. In this study the maximal percentage of variation observed between fresh and frozen plasma was not considered clinically relevant and would not be expected to lead to misinterpretation of the results [17]. Thus, this study demonstrates that in bovine plasma stored at −20°C changes in protein fractions occur in TP, ALB, and beta-2 globulin expressed as g/dL after 1 month and 6 and 12 months. Since one of the main reasons for use of bovine frozen plasma is the passive transfer of immunity for treatment of hypogammaglobulinemia in calves, the preservation of the gamma globulin fraction is essential. In our study there was no difference between the gamma globulin fraction expressed as g/dL and the percentage in fresh and thawed plasma at any time of storage studied. So bovine fresh frozen plasma seems to be suitable for treatment of hypogammaglobulinemia (failure of passive transfer of immunity) in calves when stored for 12 months at −20°C.

## 5. Conclusion

This study suggests that total gamma globulin concentration in bovine frozen plasma is stable for 12 months at −20°C. Total protein, ALB, and beta-2 fraction have significantly different concentrations when expressed as g/dL compared to prestorage. This study has shown IgG protein fraction stability in bovine fresh frozen plasma collected for transfusion; therefore, bovine fresh frozen plasma seems to be suitable for treatment of hypogammaglobulinemia (failure of passive transfer) in calves when stored for 12 months at −20°C.

## Figures and Tables

**Table 1:** Mean, median, and confidence interval 95% (95% CI) of total protein (TP) concentrations (biuret method), concentrations of albumin (ALB) and alpha-1, alpha-2, beta-1, beta-2, and gamma globulin fractions (agarose gel electrophoresis), and calculated albumin/globulin (A/G) ratios in fresh plasma samples and plasma samples stored frozen (−20°C) and then thawed, collected from 20 adult cows for transfusion purposes.

Storage time (months)	TP g/dL (95% CI)	ALB g/dL (95% CI)	Alpha-1 g/dL (95% CI)	Alpha-2 g/dL (95% CI)	Beta-1 g/dL (95% CI)	Beta-2 g/dL (95% CI)	Gamma g/dL (95% CI)	A/G
0	7 (6.8–7.1)	3.07 (2.9–3.2)	0.27 (0.24–0.30)	0.37 (0.34–0.38)	0.27 (0.26–0.28)	0.53 (0.48–0.57)	2.97 (2.8–3.06)	0.6 (0.53–0.65)

1	7.45 (6.8–7.9)	3.02 (2.8–3.1)	0.28 (0.26–0.31)	0.37 (0.33–0.41)	0.29 (0.25–0.31)	0.55 (0.50–0.60)	3.02 (2.72–3.59)	0.61 (0.53–0.70)

6	7.45 (7.1–7.8)	3.09 (2.9–3.2)	0.26 (0.23–0.29)	0.37 (0.34–0.42)	0.29 (0.27–0.30)	0.57 (0.53–0.64)	3.16 (2.87–3.33)	0.63 (0.55–0.70)

12	7.25 (7.1–7.5)	3.3 (3.1–3.4)	0.28 (0.25–0.31)	0.37 (0.36–0.40)	0.31 (0.29–0.32)	0.6 (0.53–0.70)	3.11 (2.77–3.33)	0.61 (0.54–0.68)

**Table 2:** Effects of storage time on protein parameters in bovine plasma obtained for transfusion purposes and stored frozen at −20°C for 12 months. Mean, median, *P* value (referred to Kruskal-Wallis (K-W) test and paired *t*-test or Mann-Whitney (M-W) test), and percentage of variation in total protein (TP), albumin (ALB), and beta-2 globulin fractions (agarose gel electrophoresis) at different time of storage.

Sample	Time (months)	*P* value P. *t*-test M-W	*P* value K-W	Mean/median difference	Percentage of variation %
TP	0-1	0.0108	0.0336	7–7.45 g/dL	6.4%
TP	0–6	0.0023		7–7.45 g/dL	6.4%
TP	0–12	0.0027		7–7.25 g/dL	3.5%

ALB	0-1	0.0131	0.0394	2.6–2.8 g/dL	7.6%
ALB	0–6	0.0035		2.6–2.8 g/dL	7.6%
ALB	0–12	0.0038		2.6–2.8 g/dL	7.6%

Beta-2	0–6	0.0401	0.0401	0.53–0.57 g/dL	7.5%
Beta-2	0–12	0.0230		0.53–0.60 g/dL	13.2%
